# A comparison of graph- and kernel-based –omics data integration algorithms for classifying complex traits

**DOI:** 10.1186/s12859-017-1982-4

**Published:** 2017-12-06

**Authors:** Kang K. Yan, Hongyu Zhao, Herbert Pang

**Affiliations:** 1 School of Public Health, Li Ka Shing Faculty of Medicine, The University of Hong Kong, Hong Kong, China; 2 Department of Biostatistics, Yale University, New Haven, CT USA

**Keywords:** Bayesian network, Relevance vector machine, Graph-based semi-supervised learning, Semi-definite programming (SDP)-support vector machine, Multiple data sources, Classification

## Abstract

**Background:**

High-throughput sequencing data are widely collected and analyzed in the study of complex diseases in quest of improving human health. Well-studied algorithms mostly deal with single data source, and cannot fully utilize the potential of these multi-omics data sources. In order to provide a holistic understanding of human health and diseases, it is necessary to integrate multiple data sources. Several algorithms have been proposed so far, however, a comprehensive comparison of data integration algorithms for classification of binary traits is currently lacking.

**Results:**

In this paper, we focus on two common classes of integration algorithms, graph-based that depict relationships with subjects denoted by nodes and relationships denoted by edges, and kernel-based that can generate a classifier in feature space. Our paper provides a comprehensive comparison of their performance in terms of various measurements of classification accuracy and computation time. Seven different integration algorithms, including graph-based semi-supervised learning, graph sharpening integration, composite association network, Bayesian network, semi-definite programming-support vector machine (SDP-SVM), relevance vector machine (RVM) and Ada-boost relevance vector machine are compared and evaluated with hypertension and two cancer data sets in our study.

In general, kernel-based algorithms create more complex models and require longer computation time, but they tend to perform better than graph-based algorithms. The performance of graph-based algorithms has the advantage of being faster computationally.

**Conclusions:**

The empirical results demonstrate that composite association network, relevance vector machine, and Ada-boost RVM are the better performers. We provide recommendations on how to choose an appropriate algorithm for integrating data from multiple sources.

**Electronic supplementary material:**

The online version of this article (doi: 10.1186/s12859-017-1982-4) contains supplementary material, which is available to authorized users.

## Background

Recent advancements in –omics technologies have given us an unprecedented opportunity to understand the role of genomic, epigenetic, transcriptomic features in human health and complex diseases. With the lowering of sequencing cost and the availability of different sources of –omics data, more thorough and comprehensive analysis of complex phenotypes can be achieved by integrating these diverse data sources, as a single data source is unlikely to provide a full and clear picture of human diseases. Data integration may allow us to identify patterns that become evident across different experiments, such as the identification of disease-gene association by integrating different gene networks (i.e. functional interaction network, cancer module network and gene chemical network) using gene prioritization methods [1]. Thus, there is a great need to develop powerful data integration methodologies to fully harness the potential of these high-throughput data.

The ability to integrate multiple data sources can better inform researchers about the nature of the gene networks and biological interactions involved in disease. Each genomic data source used in an integrative method gives information on a different aspect of biology, such as mutation, regulation, and expression. For now, published results have shown that the results of integrated data set can outperform individual data source. For example, Taskesen *et al.* [2] have shown that prediction of known molecular subtype of acute myeloid leukemia could be further improved by integrating gene expression and DNA-methylation profiles. Ma *et al.* [3] have proposed an effective method for the integrative analysis of DNA-methylation and gene expression in epigenetic modules. Graph and kernel methods are common ways for integrating multiple data sources for the classification of binary traits. The raw data are first mapped using graph or kernel methods to form relationships between samples before the data integration step. Graph is a natural way to depict relationships among samples with subjects denoted by nodes and their relationships denoted by edges. Multiple graph- and kernel-based data integration algorithms have been proposed, making the selection of appropriate tools difficult. Recently, there has been a community effort to identify top data integration algorithms for predicting a continuous outcome such as drug sensitivity in human breast cancer cell lines [4]. However, up till now and to the best of our knowledge, there has not been reviews comparing the performance of these algorithms for binary outcomes. There is a lack of empirical studies on how the graph- and kernel-based data integration algorithms perform on real data. Therefore, our study aims to fill this gap by providing a comprehensive comparison of their performance, in terms of various measures of classification accuracy and computation time. We want to emphasize that the purpose of this paper is not to identify the best performing algorithm based on different combinations of data sources, but to compare the performance of data integration algorithms given a fixed number of data sources at hand.

**Fig. 1:**
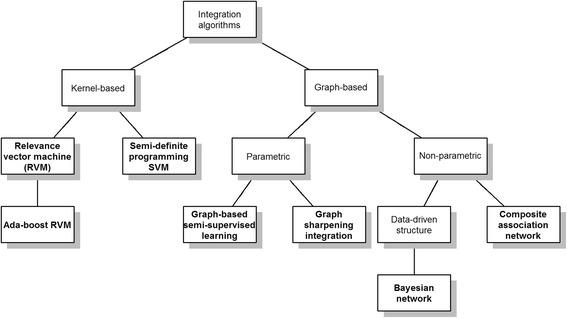
Data integration algorithms compared

We consider seven data integration algorithms, including graph-based semi-supervised learning [5], graph sharpening integration [6], composite association network [7, 8], Bayesian network [9], semi-definite programming (SDP)-support vector machine [10, 11], relevance vector machine [12, 13], and boosted relevance vector machine [14]. Figure 1 provides an overview of these seven data integration algorithms. We will briefly review these graph- and kernel-based –omics data integration algorithms. The practical usability of these tools is important, so we provide insights as to how one may choose the tuning parameters for algorithms that require them.

## Methods

### Graph-based algorithms

We first introduce the graph-based semi-supervised learning for a single network [15]. Assume a network *G* with *n* indexed nodes (1, 2, ⋯, *n*), where the first *p* nodes are labelled as binary (known status), *y*_1_, *y*_2_, ⋯, *y*_*p*_ and *y*_*i*_ ∈ {−1, 1}, and the remaining *n* − *p* unlabelled nodes will be assigned as 0 (unknown status). The main task of graph-based semi-supervised learning is to classify these unlabelled nodes utilizing the network structure related to these nodes. The symmetric weight matrix *W*, represents the connection strength between these nodes. The elements of *W* are non-negative (*w*_*ij*_ ≥ 0) which represents the degree of association, and *w*_*ij*_ = 0 means that there is no edge between node *i* and node *j*. The algorithm will generate an output function score ***f*** = (*f*_1_, *f*_2_, ⋯, *f*_*n*_)^*T*^ with two assumptions, (i) the score *f*_*i*_ should be similar with the labelled node *y*_*i*_, and (ii) the score *f*_*i*_ should be close to the score of its neighbour nodes. Then ***f*** can be inferred from the following objective function:

1 $$ \underset{\boldsymbol{f}}{\mathit{\min}}\sum \limits_{i=1}^n{\left({f}_i-{y}_i\right)}^2+c\sum \limits_{i,j=1}^n{w}_{ij}{\left({f}_i-{f}_j\right)}^2 $$

The first term, $\sum \limits_{i=1}^n{\left({f}_i-{y}_i\right)}^2$, corresponds to the squared loss function that measures the sum of squared differences between the true value *y*_*i*_ and the function score *f*_*i*_; the second term, $\sum \limits_{i,j=1}^n{w}_{ij}{\left({f}_i-{f}_j\right)}^2$, corresponds to the smoothness assumption. Here, *c* is a trade-off parameter which controls the importance of the smoothness versus loss. This objective function can be rewritten as,

2 $$ \underset{\boldsymbol{f}}{\mathit{\min}}{\left(\boldsymbol{f}-\boldsymbol{y}\right)}^T\left(\boldsymbol{f}-\boldsymbol{y}\right)+c{\boldsymbol{f}}^TL\boldsymbol{f} $$

where ***y*** = (*y*_1_, *y*_2_, ⋯, *y*_*n*_)^*T*^, and *L* is defined as the Laplacian matrix of network *G*, *L* = *D* − *W*, *D* = diag(*d*_*i*_), and *d*_*i*_ = ∑_*j*_ *w*_*ij*_. The optimal solution can be obtained by ***f*** = (*I* + *cL*)^−1^ ***y***. Then we will predict the unlabelled nodes by the median cut-off. Node will be classified as *y*_*i*_ = 1 when its function score *f*_*i*_ is closer to the median function scores of nodes labelled as 1, otherwise, node will be classified as *y*_*i*_ =  − 1.

Computation can be time-consuming and memory intensive when the dimension of *L* gets large. In reality, *L* can be very sparse, which makes it possible for the graph-based semi-supervised learning to be applied in large scaled networks.

#### Graph-based semi-supervised learning

Given a group of nodes, different data sources may have different network structures and connection strengths among these nodes. Integrating different data sources by utilizing their network structure is an intuitive way for addressing the classification problem. Based on the concept of a single network graph-based algorithm, an extension using convex optimization model can be used to combine multiple data sources [5].

Assume that we have multiple network structures for a given set of nodes, the Laplacian matrices are represented as *L*_1_, *L*_2_, ⋯, *L*_*m*_, then this integration problem can be formulated as below:

3 $$ \underset{\boldsymbol{f},\gamma }{\mathit{\min}}{\left(\boldsymbol{f}-\boldsymbol{y}\right)}^T\left(\boldsymbol{f}-\boldsymbol{y}\right)+ c\gamma \kern2.5em {\boldsymbol{f}}^T{L}_k\boldsymbol{f}\le \gamma, k=1,\cdots, m. $$

where γ is the upper bound of the smoothness function ***f*** ^*T*^ *L*_*k*_ ***f*** over all networks.

By performing Lagrange multipliers (*α*_*k*_, *η* ≥ 0), this objective function can be rewritten as following:

4 $$ \underset{\alpha, \eta }{\mathit{\max}}\underset{\boldsymbol{f},\gamma }{\mathit{\min}}{\left(\boldsymbol{f}-\boldsymbol{y}\right)}^T\left(\boldsymbol{f}-\boldsymbol{y}\right)+ c\gamma +\sum \limits_{k=1}^m{\alpha}_k\left({\boldsymbol{f}}^T{L}_k\boldsymbol{f}-\gamma \right)-\eta \gamma $$

Note that *L*_*i*_ is symmetric. This new objective function will achieve its optimal when the derivative of ***f*** equals to zero. Function scores can be solved by using $\boldsymbol{f}={\left(I+\sum \limits_{k=1}^m{\alpha}_k{L}_k\right)}^{-1}\boldsymbol{y}$.

Obviously, the function score *f* is formulated in terms of Lagrange multipliers, and the sum of all Lagrange multipliers will be constrained by parameter *c*. To solve this problem, substitute ***f*** in the objective function above, the convex optimization problem will be equivalent to a minimization problem:

5 $$ {\displaystyle \begin{array}{c}\underset{\alpha }{\mathit{\min}}\ {\boldsymbol{y}}^T{\left(I+\sum \limits_{k=1}^m{\alpha}_k{L}_k\right)}^{-1}\boldsymbol{y}\\ {}s.t.\kern1.25em \sum \limits_{k=1}^m{\alpha}_k\le c\end{array}} $$

*α*_*k*_ is treated as the weight of the network structure *G*_*k*_. The optimal function score can be obtained after solving this convex optimization problem. Network structures with zero weights will be considered as redundant, which has no contribution to the optimal function score. The prediction process will be the same as the single network using a cut-off by median.

#### Graph sharpening integration

In reality, the Laplacian matrix can be very dense and high-dimensional occasionally, which will result in longer computation time when graph-based semi-supervised learning is performed. In order to reduce the computation time and maintain or increase the current performance of graph-based semi-supervised learning, Shin *et al.* [6] proposed the graph sharpening integration method that reduces the complexity of the weight matrix in the graph-based learning algorithm. The relationship among labelled and unlabelled points described by weight matrix *W* is symmetric while it is not desirable to be all symmetric**.** That is, some edges may carry more useful information in one direction than in the opposite direction. Therefore, edges between opposite labelled points maybe unnecessary. Removing some edges in a graph structure will yield a sparser and more parsimonious graph and reduce some computational burden. Suppose a network structure with weight matrix *W*, and *w*_*ij*_ represents the edge strength from node *j* to node *i*. Firstly, edges from unlabelled nodes to labelled nodes will be removed, then edges between opposite labelled nodes will also be removed. That is, *w*_*ij*_ = 0 if node *i* is labelled and node *j* is unlabelled or nodes *i*, *j* have opposite labels. The original dense *W* is forced to stay sparse by cutting these unhelpful edges. Even after the removal of these unnecessary edges in graph sharpening algorithm, it still preserves sufficient information of the original network structure. First, no information will be lost on the labelled nodes, their influence to neighbour nodes still exists. Second, the connection information of unlabelled nodes is also preserved. So the performance should be reasonable when compared to graph-based semi-supervised learning, this can be illustrated by the results shown in Shin *et al.* [6].

In contrast to the graph-based semi-supervised learning, the weight matrix *W* in graph sharpening integration is no longer symmetric. The Laplacian matrix *L* becomes asymmetric. Considering the objective function in graph-based integration algorithm, the optimal solution can be written as

6 $$ \boldsymbol{f}={\left[\ \boldsymbol{I}+\frac{1}{2}\sum \limits_{\boldsymbol{k}=1}^{\boldsymbol{m}}{\boldsymbol{\alpha}}_{\boldsymbol{k}}\left({\boldsymbol{L}}_{\boldsymbol{k}}+{\boldsymbol{L}}_{\boldsymbol{k}}^{\boldsymbol{T}}\right)\right]}^{-1}\boldsymbol{y} $$

Similar to graph-based semi-supervised learning, *α*, the weights of the different network structures can be obtained easily from the convex optimization problem by substituting *f* in the objective function. The prediction is once again based on the median cut-off.

The algorithms we have described so far involve a tuning parameter *c*, which is a trade-off between loss of information and smoothness. This value will be determined by repeated *k*-fold cross-validation using the training set through a search based on the following values.

$$ c\in \left\{0.001,0.005,0.01,0.05,0.1,0.25,0.5,1,1.5,5,10,25,50,100\right\} $$

#### Composite association network

It is obvious that the weights assigned to the different networks in graph-based semi-supervised learning and graph sharpening integration are determined by solving a convex optimization problem. The computation will be very costly unless *L* is very sparse. The composite association network approach [7] addresses this limitation by using linear regression to obtain the weights of different data sources.

Assume that *m* associated networks with symmetric weight matrices *W*_*i*_ and that the elements of *W*_*i*_ which indicate the edge strengths are all non-negative. Let ***y*** = (*y*_1_, *y*_2_, ⋯, *y*_*n*_)^*T*^ be the label vector of nodes in the networks and element *y*_*i*_ be a binary variable, *y*_*i*_ ∈ {−1, 1}. The target network *T* is defined as the functional relationships of ***y***. *T*_*ij*_ will take one of three values.

7 $$ {T}_{ij}=\left\{\begin{array}{c}{\left({n}_{+}/n\right)}^2\kern3.25em {y}_i={y}_j=-1\kern2.5em \\ {}{\left({n}_{-}/n\right)}^2\kern3em \ {y}_i={y}_j=1\kern3.5em \\ {}\left({n}_{+}{n}_{-}/{n}^2\right)\kern2em {y}_i\ne {y}_j\kern5.5em \end{array}\right. $$

where *n*_+_/*n*_−_ is the total number of positives/negatives in label vector. The target is to integrate the *m* associated networks with weights ***α*** = (*α*_1_, *α*_2_, ⋯, *α*_*m*_)^*T*^, and the composite weight matrix is $\overline{W}=\sum \limits_{i=1}^m{\alpha}_i{W}_i$. Intuitively, in a target network *T*, pairs of positive/negative labelled nodes will have high similarity whereas pairs with a positive node and a negative node will have low similarity. The values of *T* will influence the weights of the composite association networks. The objective function will minimize the least squares error between target network *T* and composite weight matrix $\overline{W}$.

8 $$ \underset{\boldsymbol{\alpha}}{\mathit{\min}} trace\left({\left(\overline{W}-T\right)}^T\left(\overline{W}-T\right)\right) $$

Note that *trace*(*AB*) = *vec*(*A*)^*T*^ *vec*(*B*), the objective function can be rewritten as below

9 $$ \underset{\boldsymbol{\alpha}}{\mathit{\min}}{\left(\varOmega \boldsymbol{\alpha} - vec(T)\right)}^T\left(\varOmega \boldsymbol{\alpha} - vec(T)\right)\kern1.5em \varOmega =\left[ vec\left({W}_1\right),\cdots, vec\left({W}_m\right)\right] $$

The optimal solution can be obtained by setting the derivative of ***α*** equal to zero.

10 $$ \boldsymbol{\alpha} ={\left({\varOmega}^T\varOmega \right)}^{-1}\left({\varOmega}^T vec(T)\right) $$

As we mentioned above, the target network *T* only takes three values, that is *vec*(*T*) can be treated as pair-specific covariates. In our case, we specified three categorical variables: positive-positive, negative-negative and positive-negative [7]. Different from the graph based semi-supervised learning, the weight obtained with composite association network may be negative. To avoid this situation, *α*_*i*_ will be set to zero when it is negative. Average weights *α*_*i*_ = 1/*m* will overwrite the original weights when *α*_*i*_ ≤ 0 for all *i* for the association networks. In practice, a bias weight *α*_0_ will be added in ***α*** and the first column of Ω will be filled by one. *α*_0_ will be discarded when integrating the weight matrices of the association networks.

Once we obtain the composite weight matrix $\overline{W}$, we will employ the graph-based semi-supervised learning for a single network. The function scores can be solved by the formula ***f*** = (*I* + *cL*)^−1^ ***y***, where *L* is the Laplacian matrix related to weight matrix $\overline{W}$. *c* will be set to 1 for the composite association network as in the original paper by Mostafavi *et al.* [8].

#### Bayesian network

Bayesian network [9] is a probabilistic directed acyclic graphical model that composed of a set of random variables and their conditional dependencies. Nodes in a Bayesian network represent different variables and their conditional dependencies are specified via directed edges. Each node is associated with a probability function that takes a particular set of values of its parent variables as input and gives the probability of the variable represented by this node as output. The main idea of this approach is that it involves Bayesian inference, that is, the posterior probability can be computed as the product of prior probability and likelihood probability. Now we will describe the use of Bayesian network for data integration.

Suppose we have *n* samples with *m* variables *v*_1_, *v*_2_, ⋯, *v*_*m*_, which are classified into two groups and labelled as *y*, where *y* ∈ {−1, 1}, and the first *k* variables *v*_1_, *v*_2_, ⋯, *v*_*k*_ are conditionally dependent and the remaining variables are conditionally independent given *y*. With the given samples, the prior probability *p*(*y*) and the likelihood probability *p*(*v*_1_, *v*_2_, ⋯, *v*_*m*_| *y*) can be obtained directly. Then the posterior probability of *y*, denoted as *p*(*y*| *v*_1_, *v*_2_, ⋯, *v*_*m*_) can be expressed as

11 $$ p\left(y|{v}_1,{v}_2,\cdots, {v}_m\right)p\left({v}_1,{v}_2,,,\cdots, {v}_m\right)=p\left({v}_1,{v}_2,\cdots, {v}_m|y\right)p(y) $$

As the computation of *p*(*v*_1_, *v*_2_, ⋯, *v*_*m*_) can be cumbersome, an intuitive way is to use the posterior odds ratio rather than the posterior probability. Posterior odds ratio can be computed by the likelihood odds ratio and the prior odds ratio. That is,

12 $$ {Odd}_{post}=\frac{p\left(y=1|{v}_1,{v}_2,\cdots, {v}_m\right)}{p\left(y=-1|{v}_1,{v}_2,\cdots, {v}_m\right)}=\frac{p\left({v}_1,{v}_2,\cdots, {v}_m|y=1\right)p\left(y=1\right)}{p\left({v}_1,{v}_2,\cdots, {v}_m|y=-1\right)p\left(y=-1\right)} $$

$\frac{p\left(y=1\right)}{p\left(y=-1\right)}$ can be represented as prior odds ratio *Odd*_*proir*_, which explains the proportion of the two groups in the sample set. Further, considering the conditional dependencies of these variables in the structure of Bayesian network, the likelihood function can be rewritten as.

13 $$ {\displaystyle \begin{array}{c}p\left({v}_1,{v}_2,\cdots, {v}_m,y\right)=p\left({v}_1,{v}_2,\cdots, {v}_k,y\right)\times p\left({v}_{k+1},{v}_{k+2},\cdots, {v}_m,y\right)\\ {}=p\left({v}_1,{v}_2,\cdots, {v}_k,y\right)\times \prod \limits_{i=k+1}^mp\left({v}_i|y\right)\end{array}} $$

Obviously, samples with *Odd*_*post*_ > 1 will be classified as 1, otherwise −1. The larger the posterior odds ratio is, the more likely *y* will be classified as 1.

In our study, important SNPs/genes will be filtered from different data sources in the first step based on the process described by Klein *et al.* [16]. Briefly for each SNP/gene, its association with the dichotomized label will be tested and the filtered SNPs/genes that pass the Bonferroni corrected *P*-values will be included. Scores will be assigned to patients based on these filtered SNPs/genes. We discretize the scores into several bins based on their respective quartiles. Edges will be added between two nodes when their conditional correlation coefficients exceeded the threshold of 0.3. Both simple Bayesian networks and structured Bayesian networks are considered in our study. Illustrations of the four graph-based learning algorithms can be found in Additional file 1: Section A.

### Kernel-based algorithms

#### Semi-definite programming SVM

Support vector machine is a well-known kernel-based algorithm that can create hyperplane classifier by solving a quadratic program based on the kernel function and labels. The use of kernel functions provides a powerful approach to detect the nonlinear relationships in the feature space, i.e. a high-dimensional representation of numerical output variables. Its main goal is to search a linear classifier in the feature space that has the maximum margin distance between two groups. Semi-definite programming SVM [10, 11] that combines semi-definite programming framework with SVM, extends the quadratic program to multiple kernels. It is readily applicable to multiple kernel learning and makes it possible to integrate different data sources with different kernel functions.

Consider a set of kernels obtained from different data sources *κ* = {*K*_1_, *K*_2_, ⋯, *K*_*m*_}, and $K=\sum \limits_{i=1}^m{\mu}_i{K}_i$ with embedding function Φ(*x*), represented as linear combination of these kernels, the combined kernel *K* is positive semidefinite if *μ*_*i*_ ≥ 0 for *i* ∈ {1, 2, ⋯, *m*}. Thus, the *μ*_*i*_ can be considered as the linear weights of kernel *K*_*i*_. Given a set of training data *x* = (*x*_1_, *x*_2_, ⋯, *x*_*n*_) with corresponding labels ***y*** = (*y*_1_, *y*_2_, ⋯, *y*_*n*_)^*T*^, where *y*_*i*_ ∈ {−1, 1}. The objective hyperplane is *w* ^*T*^Φ(*x*) + *b* = 0, where *w* is the linear combination of kernel function corresponding to *x*_*i*_. The 1-norm soft margin SVM optimization problem can be described as follows.

14 $$ {\displaystyle \begin{array}{c}\min {\left\Vert \boldsymbol{w}\right\Vert}^2+C\sum \limits_{i=1}^n{\xi}_i\\ {}s.t.\kern0.75em {y}_i\left({\boldsymbol{w}}^T\Phi \left({x}_i\right)+b\right)\ge 1-{\xi}_i\\ {}{\xi}_i\ge 0,i=1,\cdots, n\end{array}} $$

where *C* is a penalty parameter that trades-off between margin and loss. By considering its corresponding dual problem, Schölkopf and Smola [17] proved that the weight vector could be represented as $w={\sum}_{i=1}^n{\alpha}_i\Phi \left({x}_i\right)$, where support vector ***α*** could be solved from the following equation.

15 $$ {\displaystyle \begin{array}{c}\underset{\mu_i}{\min}\underset{\boldsymbol{\alpha}}{\max }2{\boldsymbol{\alpha}}^T\boldsymbol{e}-{\boldsymbol{\alpha}}^T\mathit{\operatorname{diag}}\left(\boldsymbol{y}\right)\left(\sum \limits_{i=1}^m{\mu}_i{K}_i\right)\mathit{\operatorname{diag}}\left(\boldsymbol{y}\right)\boldsymbol{\alpha} \\ {}s.t.\kern1em trace\left(\sum \limits_{i=1}^m{\mu}_i{K}_i\right)=c\\ {}\sum \limits_{i=1}^m{\mu}_i{K}_i\succcurlyeq 0\\ {}\begin{array}{l}{\boldsymbol{\alpha}}^T\boldsymbol{y}=0\\ {}0\le \boldsymbol{\alpha} \le C\end{array}\end{array}} $$

Here *c* is a regularization parameter that controls the linear weights of the kernels and ***e*** is a vector of ones. This convex problem can be reformulated as a quadratically constrained quadratic program (QCQP) after considering its Lagrange dual problem.

16 $$ {\displaystyle \begin{array}{c}\underset{\boldsymbol{\alpha}, t}{\max }\ 2{\boldsymbol{\alpha}}^T\boldsymbol{e}- ct\\ {}s.t.\kern1em t\ge \frac{1}{r_i}{\boldsymbol{\alpha}}^T\mathit{\operatorname{diag}}\left(\boldsymbol{y}\right){K}_i\mathit{\operatorname{diag}}\left(\boldsymbol{y}\right)\boldsymbol{\alpha} \\ {}{r}_i=\sum \limits_{j=1}^m{\left[{K}_i\right]}_{jj}\\ {}\begin{array}{l}{\boldsymbol{\alpha}}^T\boldsymbol{y}=0\\ {}0\le \boldsymbol{\alpha} \le C\end{array}\end{array}} $$

This QCQP is a special form of semi-definite programming that can be solved efficiently with interior point methods [18]. The computational complexity of solving this SDP can be *O*(*mn* ^3^) in the worst case. Solving this problem results in the optimal solution for ***α*** and the optimal values for its dual variables *μ*_*i*_. Finally, the hyperplane classifier *f* = *w* ^*T*^ ***x*** + *b* will be calculated via formula $w=\sum \limits_{i=1}^n{\alpha}_iK\left({x}_i,x\right)$ where $K=\sum \limits_{i=1}^m{\mu}_i{K}_i$, and $b=-\frac{\max_{i,{y}_i=-1}{w}^T{x}_i+{\max}_{i,{y}_i=1}{w}^T{x}_i}{2}$. An unclassified *x* will be classified as 1 when *f* is positive, otherwise will be classified as −1.

In our study, *c* is set to be the training set sample size that ensures the sum of the weights equals to one and *C* is determined by grid search.

#### Relevance vector machine

Relevance Vector Machine (RVM) is a machine learning technique with an identical functional form to support vector machine (SVM), but employs Bayesian inference to obtain probabilistic results [12, 13]. Given a set of input samples ${\left\{{x}_n\right\}}_{n=1}^N$ with the corresponding output ${\left\{{y}_n\right\}}_{n=1}^N$, where *x*_*n*_ ∈ *R* ^*d*^ and *y*_*n*_ ∈ {−1, 1}. The RVM classification model can be written as a linear combination of kernel functions *k*

17 $$ Y\left(x;w\right)=\sum \limits_{i=1}^N{w}_ik\left(x,{x}_i\right)={W}^TK $$

where *W* = [*w*_1_, *w*_2_, ⋯, *w*_*N*_] and *K* = [*k*(*x*, *x*_1_), *k*(*x*, *x*_2_), ⋯, *k*(*x*, *x*_*N*_)].

Finally, *m* samples will be reserved as relevance points. The probability is calculated by the following sigmoid function:

18 $$ P\left({y}_i=1|W\right)=\frac{1}{1+{e}^{-Y\left(x;w\right)}} $$

The performance of RVM can be very similar to SVM, but RVM is more competitive than SVM in the following aspects. (i) The result of RVM is sparser than SVM and the kernel computation time can be largely reduced; (ii) RVM can provide probabilistic prediction for classification problems by returning the class probabilities; (iii) RVM does not require the specification of a loss parameter; and (iv) Kernel function in RVM is more flexible without the Mercer’s condition [19] restriction.

Assume that *k* different associate data sources with a corresponding outcome *Y*, where *Y* = (*y*_1_, *y*_2_, ⋯, *y*_*n*_)^*T*^ and *y*_*i*_ ∈ {−1, 1}. For each data source, an individual RVM model will be generated with the corresponding kernel matrix, i.e. radial basis function kernel. Denote *P*_1_, *P*_2_, ⋯, *P*_*k*_ as the *k* sets of probability prediction results from multiple RVM models, where *P*_*i*_ is an *n* × 1 vector. The final probability is given by

19 $$ \overline{P}=\left({P}_1+{P}_2+\cdots +{P}_k\right)/k={\left({p}_1,{p}_2,,,\cdots, {p}_n\right)}^T $$

Note that *p*_*i*_ is the probability of *y*_*i*_ = 1. The cut-off point should be 0.5, which means sample will be classified as 1 when *p*_*i*_ > 0.5. The greater *p*_*i*_ is, the higher the chance that *y*_*i*_ will be classified as 1.

#### Ada-boost RVM

Ada-Boost is a machine learning algorithm that can combine different types of learners to improve the final performance. The final classifier is the weighted sum of many weak learners. When combined with RVM [14], it will follow the following steps. Assume a set of training samples ${\left\{{x}_n\right\}}_{n=1}^N$ with the corresponding output ${\left\{{y}_n\right\}}_{n=1}^N$, where *x*_*n*_ ∈ *R* ^*d*^ and *y*_*n*_ ∈ {−1, 1}. Let *w*_*i*_ = 1/*N* denote the weights of the training samples. First, train an RVM learner on *n* random samples selected from the training set without replacement, denoted as *RVM*_*t*_, then calculate the weighted error for misclassification on the training samples in the *t*_*th*_ iteration by formula ${\varepsilon}_t=\sum \limits_{i=1}^N{w}_i$. If *ε*_*t*_ ≥ 0.5, jump to the next iteration; otherwise, set the weight of this learner *RVM*_*t*_ equal to ${\alpha}_t=\frac{1}{2}\ln \left(\frac{1-{\varepsilon}_t}{\ {\varepsilon}_t}\right)$, then the final model will update as *RVM*_*final*_ = *RVM*_*final*_ + *α*_*t*_ *RVM*_*t*_. The weights of samples will be updated as

20 $$ {w}_i=\left\{\begin{array}{c}{w}_i{e}^{\alpha_t}\kern2.5em if\ {RVM}_t\left({x}_i\right)\ne {y}_i\\ {}{w}_i{e}^{-{\alpha}_t}\kern1.75em if\ {RVM}_t\left({x}_i\right)={y}_i\end{array}\right. $$

The new weights *w*_*i*_ should be normalized such that $\sum \limits_{i=1}^N{w}_i=1$ before moving to the next iteration. After *T* iterations, the final model can be represented as *RVM*_*final*_ = ∑_*j*_ *α*_*j*_ *RVM*_*j*_, where *ε*_*j*_ < 0.5 .

As RVM is computationally intensive, using Ada-boost for RVM could address the problem of large-scale learning and lower the computational cost. Its main concept is to sample many small training sets from the original training set and then each model is trained with a smaller training set and thus lowering the computational cost. As a sufficient number of base models are generated, most of the distinct aspects of the complete training set can be captured and represented in the final combined model. It is necessary to determine an appropriate resampling size and the maximum number of iterations when utilizing the Ada-boost RVM algorithm. A range of values for resampling size and the number of iterations are evaluated by 5-fold cross validation. We search the appropriate resampling size and maximum iteration number from a search over.

$$ resampling size\in \left\{0.2N,0.4N,0.6N,0.8N\right\}, iteration\in \left\{1,5,10,20,30.\right\} $$

where *N* is the training set sample size. The pseudo code for Ada-boost RVM can be found in Additional file 1: Section B.

### Performance measure

To evaluate the performance of different data integration algorithms, we employ three measurements in our study: accuracy rate, *F*1 score (also called the F-measure) and the Area Under the receiver operating characteristic (ROC) Curve (AUC). Accuracy rate measures the percentage of entities which are correctly classified. *F*1 score combines the precision and recall rates in classification problems, and can be calculated as the harmonic mean of precision and recall rates. Given a binary classification problem with *P* positive and *N* negative entities, the predicted and true labels can form a 2 × 2 confusion matrix. Four different values: true positive *tp*, false positive *fp*, false negative *fn* and true negative *tn*, can be calculated from this table. Sensitivity and specificity are defined as

$$ sensitivity=\frac{tp}{P}, specificity=\frac{tn}{N}, $$

the accuracy rate and *F*1 score can be calculated as

$$ accuracy=\frac{tp+ tn}{tp+ fp+ tn+ fn},F1=\frac{2 tp}{2 tp+ fp+ fn}. $$

ROC curve captures the sensitivity as a function of (1-specificity). It illustrates the overall performance of a binary classifier by varying the discrimination threshold. The AUC has a value between 0 and 1. A value of 1 implies that the algorithm has a perfect classification while a value of 0.5 suggests that the algorithm is no better than a random guess.

These three performance measures are determined over 200 runs. 95% confidence intervals, calculated based on percentile bootstrap, are used to assess the variability of the algorithms. Computation time will also be considered as an evaluation factor in our study. It is clocked based a desktop running with R version 3.2.3 using an Intel Core i7 3.60 GHz PC with 16 GByte of memory. The computation time is based on integration of three different data sources that only include the model training session. Computation time of calculating the weight matrix and kernel matrix, and the filtering of SNPs/genes in the Bayesian network model are excluded.

## Data sets

Data from hypertension and cancer are used to evaluate and compare the seven data integration algorithms. Hypertension is known as the leading cause of cardiovascular mortality in the world [20]. Moreover, cancer and heart disease are the leading causes of death. Our understanding of these complex diseases from different angles of biology can be improved with the availability of multi-omics data integration algorithms. The Genetic Analysis Workshop (GAW) 19 data set was evaluated in our study, which includes data on genotypes, gene expression, and clinical data (including blood pressure and covariates such as smoking status and age). For this family data, there are 312 patients with normal blood pressure, and 305 pre-hypertension and hypertension subjects from 17 families.

Ovarian cancer and breast cancer are the two cancers evaluated in our study, which can be available from The Cancer Genome Atlas (TCGA) project [21, 22]. Four different data sources in the ovarian cancer data set, including gene expression, miRNA expression, protein expression, and methylation, are included in our analysis. There are 85 patients with lymphatic invasion and 50 without lymphatic invasion outcomes which characterize the aggressiveness of ovarian cancer. Four different data sources in the breast cancer data set, including RNASeq, miRNA expression, protein expression, and methylation, are included in our analysis. There are 351 patients with positive ER status and 102 subjects with negative ER status. The GAW 19 and TCGA are two of the largest publicly available heart disease and cancer databases with the availability of multi-omics data. Table 1 describes the data sets considered in our study.

**Table 1:** Data sets used for evaluating the data integration algorithms

Data Set	Sample Size	Data Source	Platform	Numbers of Features
GAW 19	617	Genotypes	lllumina Infinium Beadchips	440,762
		Gene Expression	lllumina Sentrix Human-6 Expression BeadChips	20,634
		Clinical Covariates	Clinical Data	2
Ovarian	135	Gene Expression	Agilent G4502A	17,814
		miRNA Expression	Agilent Human miRNA 8x15K	799
		Protein Expression	Reverse phase protein array	176
		Methylation	HumanMethylation 27	24,981
Breast	453	RNA SeqV2	Illumina HiSeq	20,531
		miRNA Expression	Agilent Human miRNA 8x15K	1046
		Protein Expression	Reverse phase protein array	166
		Methylation	HumanMethylation 450	396,065

The impact of imbalance data sets on the performance of the seven algorithms compared has also been investigated by real data simulation. In this simulation, we consider three additional situations, a more imbalanced and a more balanced breast cancer data sets by sampling without replacement, resulting in positive ER status against negative ER status ratios of 5:1 and 5:2, respectively. The breast cancer data set is chosen because it is the most imbalanced and has a relatively large sample size.

## Results

In this section, we present the empirical assessment of the seven data integration algorithms. The results compared in the following section are based on (1) Pearson correlation matrix; (2) simple Bayesian network and (3) radial basis function kernel with a scaling parameter sigma that is determined by grid search using 5-fold cross validation in the training set. The reasons are as following: In our study (1) Spearman’s rank correlation matrix and Pearson correlation matrix are used as weight matrix in graph-based semi-supervised learning, graph sharpening integration, and composite association network, the negative elements in the two correlation matrix will set to zero as weight matrix should be non-negative. The performance of Spearman’s rank correlation matrix is only slightly better than Pearson correlation matrix in most cases for the graph-based algorithms while its computational complexity is *O*(*n* ^2^ log *n*), which may become prohibitive for larger sample sizes; (2) Simple Bayesian network and structured Bayesian network are compared in our study. The performance of simple Bayesian network and structured Bayesian network are similar but structured Bayesian network leads to infinite odds ratio frequently due to small sample size; (3) Linear kernel and radial basis function kernel are tested in kernel based algorithms. Radial basis function kernel performs better than linear kernel in kernel-based algorithms in the three data sets investigated.

### Performance comparisons

**Fig. 2:**
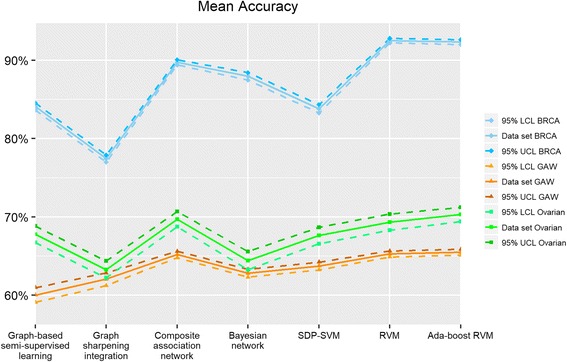
Mean accuracy of seven integration algorithms. BRCA represents breast cancer data set, GAW represents GAW 19 data set, and Ovarian represents ovarian cancer data set. “95% LCL” is the abbreviation of “95% lower confidence limit” and “95% UCL” is the abbreviation of “95% upper confidence limit”

**Fig. 3:**
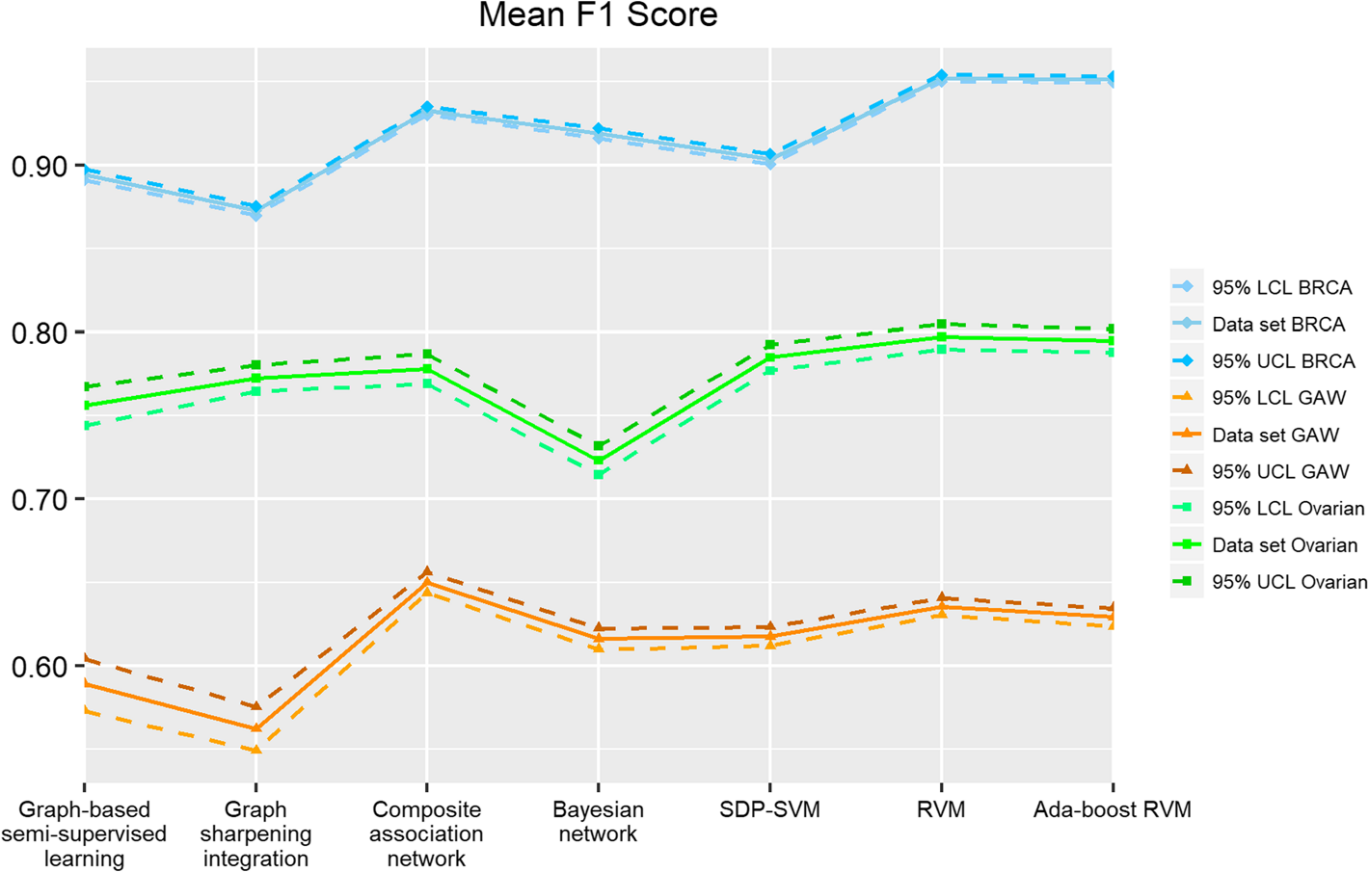
Mean F1 score of seven integration algorithms. BRCA represents breast cancer data set, GAW represents GAW 19 data set, and Ovarian represents ovarian cancer data set. “95% LCL” is the abbreviation of “95% lower confidence limit” and “95% UCL” is the abbreviation of “95% upper confidence limit”

**Fig. 4:**
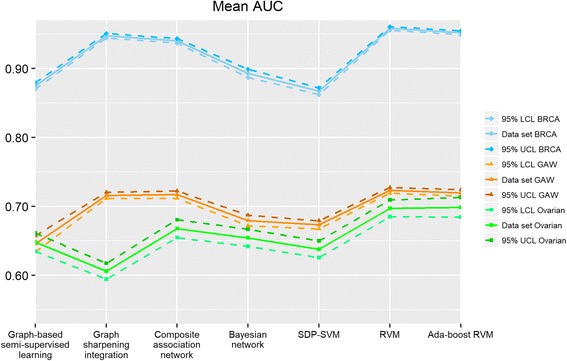
Mean AUC score of seven integration algorithms. BRCA represents breast cancer data set, GAW represents GAW 19 data set, and Ovarian represents ovarian cancer data set. “95% LCL” is the abbreviation of “95% lower confidence limit” and “95% UCL” is the abbreviation of “95% upper confidence limit”

For the two cancer data sets, we separate the data into training and testing samples, where 75% samples are randomly selected as the training set and the remaining 25% are used to evaluate the performance of the seven algorithms. For the GAW 19 data set, “Leave-cluster-out cross-validation” [23] was employed. At each iteration, 12 families will be selected as the training set and the remaining 5 families will be used as the test set. We repeat this 200 times. Figures 2, 3 and 4 show the mean accuracy, mean F1 score and mean AUC of different integration algorithms with GAW 19, ovarian and breast cancer data sets.

#### Graph-based algorithms

First, we present the results of four graph-based algorithms. As described in the materials and methods section, the difference between graph-based semi-supervised learning and graph sharpening integration is the sparseness of the weight matrix. Compared to graph-based semi-supervised learning, the graph sharpening integration still performs reasonably well with sparser weight matrices obtained from the removal of undesirable edges in network structures. However, the performance of graph sharpening integration may not be as stable which is illustrated with the three data sets. Graph sharpening performs better than graph-based semi-supervised learning with the GAW 19 data set (62.1% mean accuracy rate against 60.0%) while it performs slightly worse than graph-based semi-supervised learning with ovarian and breast data set (63.3% mean accuracy rate compared to 66.7% in ovarian and 77.5% mean accuracy rate compared to 84.1% in breast). For Fig. 2, we can observe that the confidence interval of simple Bayesian network is slightly wider than other graph-based algorithms even though the mean accuracy rates of the various graph-based algorithms are similar for the GAW 19 data set. This indicates that simple Bayesian network has a larger prediction variation than other graph-based algorithms. Composite association network usually performs better than all of the other graph-based algorithms in terms of accuracy rate, F1 score and AUC with the advantage that it only requires solving one linear regression problem. Meanwhile, it is quite stable when considering the variability of these graph-based algorithms.

#### Kernel-based algorithms

The performance of kernel-based algorithms is usually better than graph-based algorithms, while the kernel-based model is more complex and requires longer computation time due to the need to generate the hyperplane classifier. In semi-definite programming SVM, different combinations of the two tuning parameters *c*, *C* may lead to long computation time in solving the QCQP. In our study, we found that it is particularly true when *C* is less than one. RVM and Ada-boost RVM are probabilistic models, which can return probability predictions but require longer computation time when compared with semi-definite programming SVM. It is observed that Ada-boost RVM can achieve good performance with our data sets when resampling size is set to 40% or 60% of the training sample size and maximum iteration number is set to 5 or 10.

It can be seen that semi-definite programming SVM has larger variation and lower performance when compared to RVM and Ada-boost RVM. The performance of RVM and Ada-boost RVM varies in the three data sets, which make it difficult to compare these two algorithms. But the difference of mean accuracy between RVM and Ada-boost RVM is very small.

### Imbalanced data simulation

Additional file 1: Section C presents the mean accuracy, mean F1 score and mean AUC of different integration algorithms in three simulated imbalanced data sets. Among the four graph-based algorithms, the performance of composite association network and Bayesian network is less influenced by imbalanced data. The imbalanced data simulation also suggests that composite association network usually outperforms Bayesian network. The performance of RVM and Ada-boost RVM are better and more stable in the imbalanced data simulations comparing to other graph-based or kernel-based algorithms. While for SDP-SVM, its performance is affected by the imbalanced data sets.

### Computation time

Table 2 compares the average computation time (in seconds) in training the model of the seven integration algorithms with three different data sources. The sampling size of Ada-boost RVM in this part will be 40% of training size and maximum iteration number set to 10. In general, the computation time of graph-based algorithms is less than that of kernel-based algorithms in our study. Although the computation time of Bayesian network is the fastest, it requires a filtering step of SNPs/genes that is computationally costly when number of variables (i.e. SNPs/genes) gets larger. The second fastest algorithm is composite association network that only requires solving a linear regression problem. Network structure sparsity through sharpening reduces the computation time of graph sharpening integration by more than one-half of graph-based semi-supervised learning. The computation time of semi-definite programming SVM is highly dependent on the time needed to solve the QCQP. It may require more than 20 min to train the semi-definite programming SVM model in some scenarios. Training time of RVM and Ada-boost RVM is quite expensive as their computational complexity is *O*(*n* ^3^). Additional iterations of boosting procedure in Ada-boost RVM requires more time than RVM when sample size is small, 23.19 s for Ada-boost RVM against 10.47 s for RVM with 100 training samples. While the computation time of Ada-boost RVM can be largely reduced as training sample size increases when compare to RVM. It is nearly 1 minute less than RVM when sample size reaches 400.

**Table 2:** Average computation time (in seconds) of different integration algorithms with different training sizes

Integration Algorithms	Training Size 100	Training Size 400
Graph-based semi-supervised learning	0.127	4.148
Graph sharpening integration	0.052	1.943
Composite association network	0.007	0.052
Bayesian network	0.002	0.004
Semi-definite programming – SVM	12.553	28.186
Relevance vector machine	10.471	368.455
Ada-boost relevance vector machine	23.190	306.172

## Discussion

In this paper, we conducted a comprehensive comparison study of seven graph- and kernel-based data integration algorithms of subject classification using GAW 19, ovarian cancer and breast cancer data sets. From the results, we observed that the kernel-based algorithms usually perform better than graph-based algorithms, but require longer computation time. On the other hand, the graph-based algorithms require less computation time, while the performance is not as good overall.

Graph-based semi-supervised learning and graph sharpening integration involve some tuning parameters, which can be selected via *k*-fold cross validation in the training sample. In our study, we observed that graph sharpening integration could lead to average weights frequently and more variable results since the sharpening may also remove important information. Moreover, in our study, graph sharpening integration tend to have a higher mean AUC score than graph-based semi-supervised learning even though the mean accuracy rate is lower, this indicates that the prediction can be improved for non-median cut-off. Bayesian network is very sensitive to noise, inappropriate bins setting and small sample size will result in infinite odds ratio. To avoid these situations, one should adjust the bin selection to make sure that sufficient samples are contained in each bin and using simple Bayesian network instead of structured model when sample size is small. The performance of composite association network is in general very good and stable. It assigns weights to different data sources by minimizing the least square error between target network and composite weight matrix, then predict via the combined weight matrix. This unique feature makes its training process simpler than other graph-based algorithms. We can conclude that employing the composite association network may be a good choice to integration different data sources when considering among graph-based algorithms.

Kernel-based algorithms may have better performance than graph-based algorithms, but they usually require longer training time. In our study, we observe that the semi-definite programming SVM is very sensitive to outliers which leads to larger variations than RVM and Ada-boost RVM. The computation time for solving QCQP is largely dependent on the penalty and regularization parameters. This explains both the computationally intensive nature as well as the large variation seen in running the semi-definite programming SVM. RVM performs well and can return with a probabilistic prediction result but generally requires longer computation time as training sample grows. Ada-boost RVM also requires the determination of an appropriate resampling size and the number of iterations. Table 3 gives a brief summary of the different integration algorithms.

**Table 3:** Comparison of different data integration algorithms

Integration Algorithms	Computation Time	Stability	Characteristics
Graph-based semi-supervised learning	Low	Medium	Tuning parameter; performance can be poor sometimes
Graph sharpening integration	Low	Low	Tuning parameter; average weights frequently occur
Composite association network	Low	High	Average weights occur when all weights are negative
Bayesian network	Low	Low	Bins selection and training sample size affect performance
Semi-definite programming SVM	Medium	Low	Two tuning parameters; *C* is very sensitive to outliers
Relevance vector machine	High	High	Long training time; Probabilistic result
Ada-boost relevance vector machine	High	Medium	Resampling size and iteration can be hard to determine

The rationale for choosing these seven algorithms in our study is that these algorithms preserve data specific properties and can integrate data of different scales. Each data source will be transformed into an intermediate form, like a graph or kernel matrix. Graph-based integration, is a natural way to reveal the relationship among samples and it is less computationally intensive. For kernel-based integration, it is good at detecting nonlinear relationships between samples. There are other categories of integration algorithms such as the concatenation-based integration that combines multiple data sources as one large input matrix before analysis. The algorithms for this type of integration include LASSO regression and, elastic-net regression [24].

## Conclusions

**Fig. 5:**
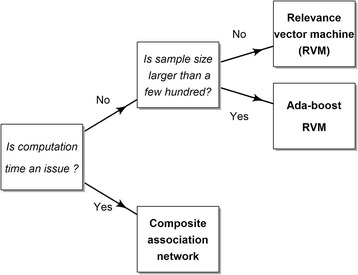
Data integration algorithms decision tree

From the analysis of the seven integration algorithms with three different data sets, the empirical results demonstrate that composite association network, relevance vector machine and Ada-boost RVM are the better performers and are less influenced by imbalanced data. No tuning parameters are required for composite association network while bins setting are needed for Bayesian network. The impact of imbalanced data on graph-based semi-supervised learning and graph sharpening integration is more obvious, especially for graph sharpening. While there is no clear indication as to which integration algorithm is superior in every situation, graph-based composite association network, relevance vector machine, and Ada-boost RVM are the better algorithms relative to other data integration algorithms in its class. They are comparable in accuracy rates but differ in computation time and form of prediction result. If time is the key issue, we would recommend composite association network, which can provide a reasonable data integration prediction in a timely manner. If someone wants a probabilistic prediction result, we would recommend relevance vector machine for small sample size and Ada-boost relevance vector machine for large sample size, for example exceeding 300 samples when setting resampling size to 40% of the training size and maximum iteration number to 10. Our recommendation can be illustrated in a decision tree in Fig. 5. In future studies, researchers may develop an ensemble classifier by utilizing a combination of the compared algorithms as this may lead to more accurate results.

## Additional file

Additional file 1: **Section A.** Graph-based integration algorithms. **Section B.** Pseudo Code for Ada-boost RVM. **Section C.** Imbalanced Data Simulation. (PDF 500 kb)
