# Supplementary material for: A comparison of graph- and kernel-based –omics data integration algorithms for classifying complex traits
Source: BMC Bioinformatics. 2017 Dec 6;18:539. doi: 10.1186/s12859-017-1982-4 (PMC6389230; doi:10.1186/s12859-017-1982-4)
Supplement: Supplementary file 1 — Section A. Graph-based integration algorithms. Section B. Pseudo Code for Ada-boost RVM. Section C. Imbalanced Data Simulation. (PDF 500 kb) [file 12859_2017_1982_MOESM1_ESM.pdf]

BMC Bioinformatics- Supplementary Materials

A comparison of graph- and kernel-based –omics data integration algorithms for classifying complex traits

Kang K. Yan[1], Hongyu Zhao[2], Herbert Pang[1, *]

Contents | Page

A) Graph–based integration algorithms 1-2
B) Pseudo Code for Ada-boost RVM 3
C) Imbalanced Data Simulation 4-6

A) Graph-based integration algorithms

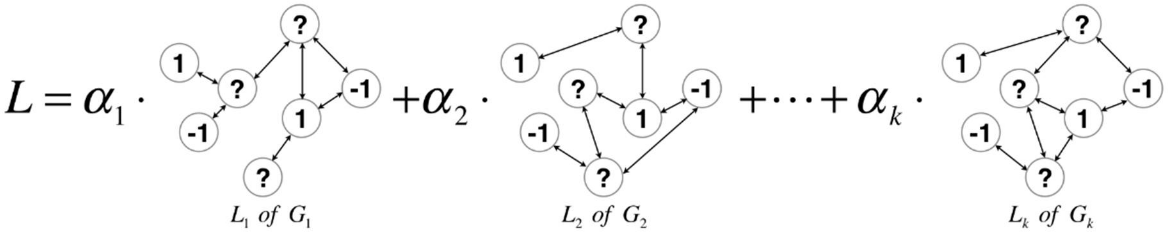

a. Graph-based semi-supervised learning

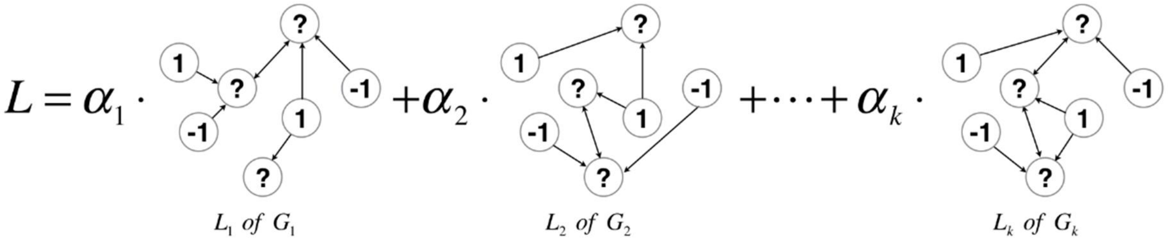

b. Graph sharpening integration

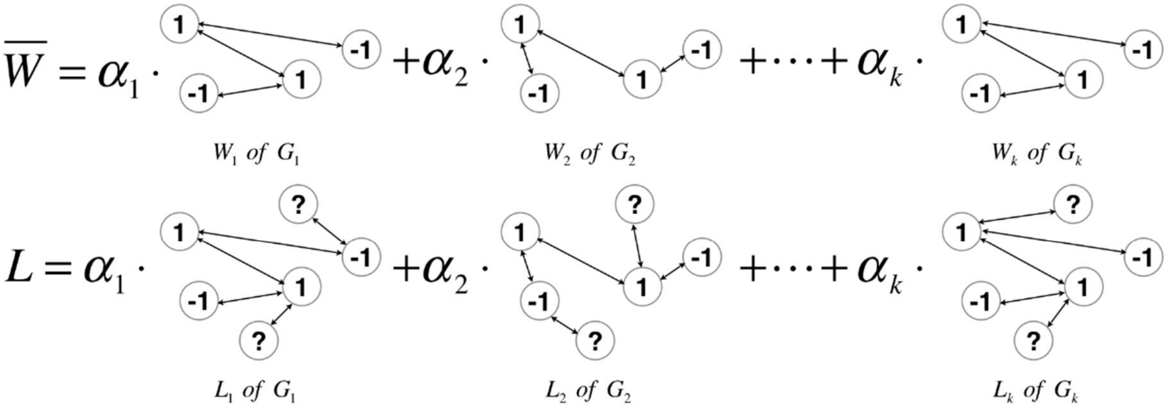

c. Composite association network

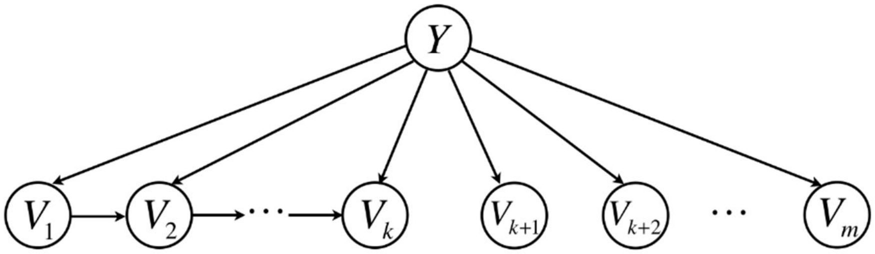

d. Bayesian network

Graph-based integration algorithms. a)Graph-based semi-supervised learning. ‘1’ or ‘-1’, indicates the binary outcome status, while ‘?’ represents unknown status. The integrated Laplacian matrix can be represented as the linear combination of these individual graph Laplacian L_k . b)Graph sharpening integration. Edges from unlabelled to labelled or between opposite labelled nodes will be removed before integration. c)Composite association network. The weights of different networks will be obtained from the nodes with known status, and the prediction procedure follows the graph-based semi-supervised learning. d)Bayesian network. This is the network structure of Bayesian network classifier. Here, Y represents the class (i.e. binary outcome, 1/-1), and $V_1, V_1 \cdots V_m$ correspond to different feature variables, the first k variables are conditionally dependent and the remaining variables are conditionally independent.

B) Pseudo Code for Ada-boost RVM

Ada-boost RVM

Input:

A set of training samples $\{x_n\}_{n=1}^{N}$ with corresponding output $\{y_n\}_{n=1}^{N}$, where $x_n \in R^d$ and $y_n \in \{-1,1\}$;

Iteration number, T;

Resampling size, n, $n < N$;

Initialize:

The weights of the training samples, $w_i = 1/N \;\; for \;\; i = 1,\cdots,N$

Loop:

For $t = 1,\cdots,T$

(1). Sampling n samples from the weighted training set without replacement

(2). Training a RVM model on the n samples, denotes as RVM_t

(3). Calculate the weighted error of RVM_t : $\varepsilon_t = \sum_{i=1}^{N} w_i(t),\; if RVM_t(x_i) \neq y_i$

(4). If $\varepsilon_t \geq 0.5$, jump to the next iteration, otherwise set the weight of this learner

$$RVM_t: \; \alpha_t = \frac{1}{2}\ln\left(\frac{1-\varepsilon_t}{\varepsilon_t}\right)$$

(5). Update the weights of training samples:

$$w_i = \begin{cases} w_i e^{\alpha_t} & if\; RVM_t(x_i) \neq y_i \\ w_i e^{-\alpha_t} & if\; RVM_t(x_i) = y_i \end{cases}$$

Normalize the new weights such that $\sum_{i=1}^{N} w_i = 1$

End

Output:

$$RVM_{final} = \sum_j \alpha_j RVM_j \qquad where\; \varepsilon_j < 0.5$$

C) Imbalanced Data Simulation

1) Mean accuracy of seven integration algorithms of different simulated balance-ness ratio from real data.

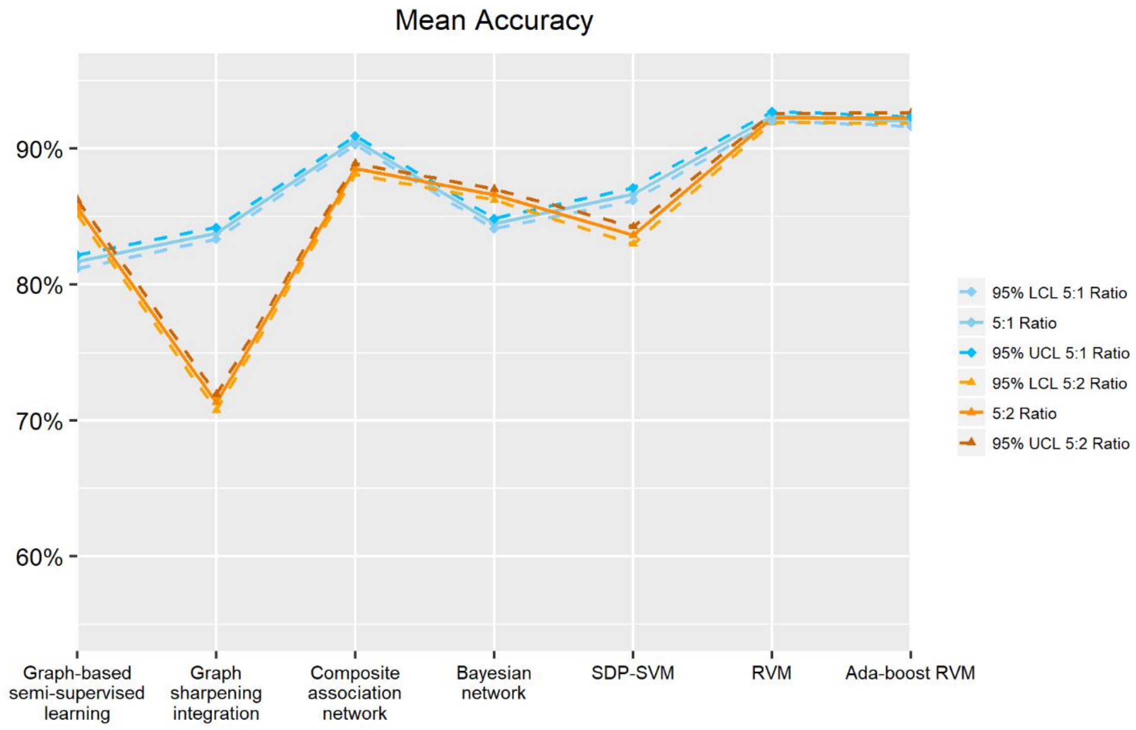

5:1 Ratio represents the cases of positive ER status are 5 times as many as the ones of negative ER status and 5:2 Ratio represents there are 2.5 times as many the cases of positive ER status as the ones of negative ER status in the simulated breast cancer data set. “95% LCL” is the abbreviation of “95% lower confidence limit” and “95% UCL” is the abbreviation of “95% upper confidence limit”.

2) Mean F1 score of seven integration algorithms of different simulated balance-ness ratio from real data.

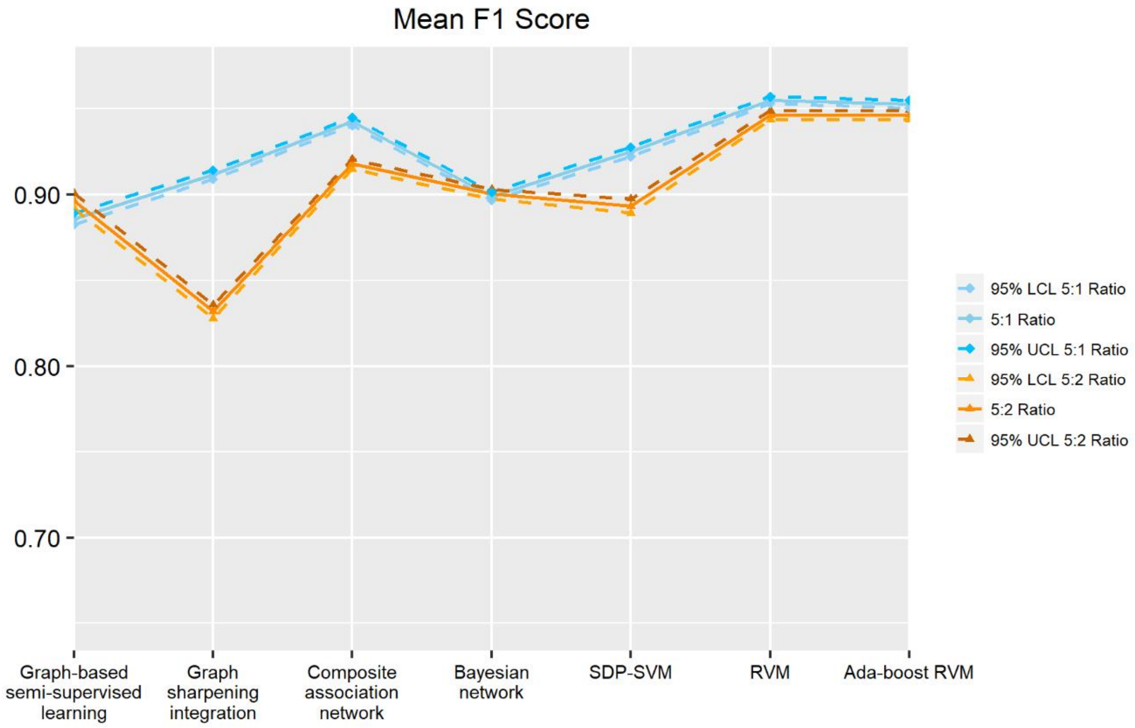

5:1 Ratio represents the cases of positive ER status are 5 times as many as the ones of negative ER status and 5:2 Ratio represents there are 2.5 times as many the cases of positive ER status as the ones of negative ER status in the simulated breast cancer data set. “95% LCL” is the abbreviation of “95% lower confidence limit” and “95% UCL” is the abbreviation of “95% upper confidence limit”.

3) Mean AUC of seven integration algorithms of different simulated balance-ness ratio from real data.

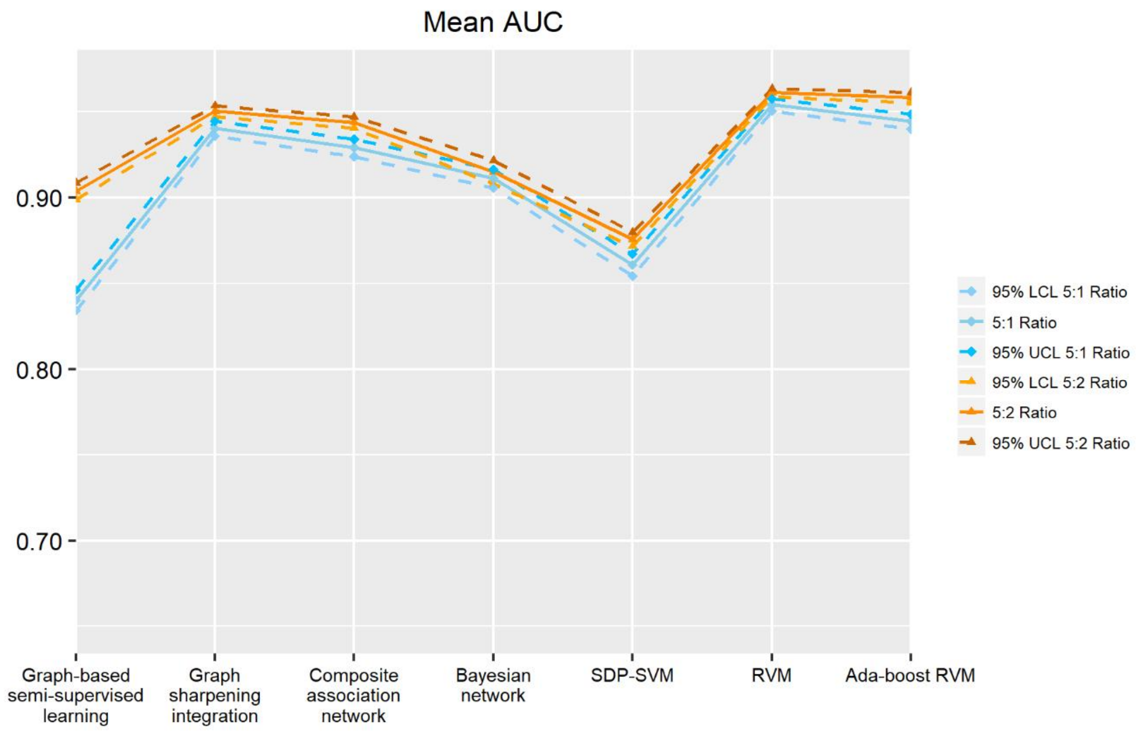

5:1 Ratio represents the cases of positive ER status are 5 times as many as the ones of negative ER status and 5:2 Ratio represents there are 2.5 times as many the cases of positive ER status as the ones of negative ER status in the simulated breast cancer data set. “95% LCL” is the abbreviation of “95% lower confidence limit” and “95% UCL” is the abbreviation of “95% upper confidence limit”.
